# Kidney Inflammation, Injury and Regeneration 2020

**DOI:** 10.3390/ijms22115589

**Published:** 2021-05-25

**Authors:** Patrick C. Baer, Benjamin Koch, Helmut Geiger

**Affiliations:** Division of Nephrology, Department of Internal Medicine III, University Hospital, Goethe-University, 60596 Frankfurt, Germany; b.koch@med.uni-frankfurt.de (B.K.); h.geiger@em.uni-frankfurt.de (H.G.)

The kidneys play a vital role in the basic physiological functions of the body. Kidney dysfunction impairs these physiological functions and can lead to a wide range of diseases. Damage to the kidney cells can be caused by a variety of ischemic, toxic or immunological complaints that lead to inflammation and cell death, which can lead to organ damage and, ultimately, complete failure. Although the mechanisms underlying acute kidney injury (AKI) and chronic kidney disease (CKD) are quite distinct, clinical evidence suggests that the two conditions are inextricably interconnected [1]. AKI and CKD, regardless of the underlying cause, have inflammation and activation of the immune system as the common underlying mechanisms. Inflammation, a process aimed, in principle, at detecting and fighting harmful pathogens, is, therefore, a major pathogenic mechanism for both AKI and CKD [1]. While the kidney has the remarkable ability to regenerate after an acute injury and can recover completely, depending on the type of kidney lesion, the options for clinical interventions are currently limited to fluid management and extracorporeal kidney support. However, persistent chronic inflammation can trigger renal fibrosis and chronic kidney disease. The investigation of the molecular mechanisms involved in each individual injury is currently insufficiently understood.

In this context, we started a forum for the publication of new results on kidney inflammation, injury and regeneration, as well as for reviewing and discussing existing studies from this interesting research area. In 2019, we initiated the first edition of the Special Edition “Kidney Inflammation, Injury and Regeneration” with 29 articles [2]. The focus of this first edition was more on a summary of current results (represented by 17 review articles), along with 12 original articles from the current research. In the second Special Edition, presented here, the focus is now more on the current research results mainly from in vivo studies. This issue is accompanied by five review articles summarizing the current results on various nephrological diseases or issues. In this current Special Edition, thirteen original research articles are presented: twelve in vivo studies in a murine or rat model and one in vitro study [3]. Seven studies show results from AKI models [4,5,6,7,8,9,10], five from fibrosis models (or CKD models) [9,11,12,13,14] and two from a transplant rejection model [4,15] (two studies used two different in vivo models).

Steines and coworkers demonstrated that intrarenal tertiary lymphoid organs are sites of humoral immune activation within allografts during chronic rejection and that anti-B-cell activating factor treatment can hinder the formation of tertiary lymphoid organs in allografts [15]. The authors hypothesized that inhibition of the local alloresponses in chronic rejection with an anti-B-cell activating factor antibody represents a potential benefit to kidney transplant patients. Others evaluated the effects of a novel human fusion recombinant protein in two representative kidney inflammatory models: a renal ischemia-reperfusion model (an AKI model) and an allogeneic kidney transplant model [4]. This study shows that targeting with that novel protein offers a good microenvironment profile to protect the ischemic process in the kidney and to prevent kidney rejection.

Another study using a renal ischemia–reperfusion (IR) model showed that preconditioning with cilastatin, a specific inhibitor of renal dehydrodipeptidase-1, attenuates renal IR injury via activation of the main hypoxia factor. The authors confirmed this effect through in vitro studies with immortal tubular epithelial cells [10]. Others used an IR model to show that the NOX1-selective inhibition attenuates kidney IR injuries via the downregulation of oxidative stress-mediated kinase signaling [8] or to investigate the autophagy dynamics during an IR injury, a potential treatment strategy due to removing damaged cells, macromolecules and organelles [6]. The effect of growth differentiation factor 15 (GDF15) was investigated in a murine model of anti-glomerular basement membrane glomerulonephritis [7]. The study showed that GDF15 is required for the regulation of T-cell chemotactic chemokines in the kidneys and demonstrated the protective effects of GDF15. The study revealed a novel mechanism limiting the migration of lymphocytes to the site of inflammation during glomerulonephritis [7]. The findings of Nežić and coworkers investigated the molecular mechanism involved in the reno-protective effects of simvastatin in an endotoxin-induced AKI model [5]. The study indicated that simvastatin, a well-known lipid-lowering medication, has cytoprotective effects on induced tubular apoptosis, mediated by the upregulation of cell survival molecules and inhibition of the mitochondrial proteins. Therefore, the authors hypothesized that simvastatin has significant cell-protective effects in septic AKI [5].

Leong and coworkers showed that cyclophilin A, a damage-associated molecular pattern, promoted inflammation and acute kidney injury in a renal IR model but did not contribute to inflammation or interstitial fibrosis in a model of progressive kidney fibrosis (unilateral ureteric obstruction (UUO)) [9]. Other studies showed the effects of different proteins/peptides against UUO-induced renal injury, inflammation and fibrosis. The 20-amino acid peptide ND-13 protects against UUO-induced damage and is, therefore, a potential new therapeutic approach to prevent renal diseases [14]. Furthermore, the effects of verteporfin on UUO-induced renal tubulointerstitial inflammation, fibrosis and transforming growth factor-β1 regulation were investigated. The study showed that verteporfin decreases the UUO-induced increase in tubular injury, inflammation and extracellular matrix deposition in mice [12]. Son and coworkers investigated the attenuating effects of dieckol on hypertensive nephropathy in spontaneously hypertensive rats and hypothesized that dieckol could be beneficial for decreasing hypertensive nephropathy by decreasing EMT and renal fibrosis [11].

Others investigated the role of xanthine oxidase (XO) in CKD progression associated with hypercholesterolemia [13]. The authors used a murine model of uninephrectomy to induce CKD, in addition to a high-cholesterol diet with a XO inhibitor, and, also, evaluated the results in an in vitro model using immortal tubular epithelial cells. The study clearly showed that XO inhibition exerts reno-protective effects and identifies XO as a novel therapeutic target for hypercholesterolemia-associated kidney injury [13]. Finally, one in vitro study using cisplatin-injured primary tubular epithelial cells focused on the decisive role of the Lipocalin-2 iron load for its pro-regenerative functions [3]. The study detected a positive correlation between the total iron amounts in tubular epithelial cells and cellular proliferation. In conclusion, it was hypothesized that macrophage-released Lipocalin-2-bound iron is provided to tubular epithelial cells during toxic cell damage, whereby the injury is limited and recovery is favored [3].

In addition, five interesting review articles were included in this Special Edition summarizing the current state of knowledge of the treatment of IgA nephropathy [16], the role of endocan in kidney diseases [17] and the influence of inflammation on anemia in CKD patients [18]. It also discusses the mechanism of kidney injury in preeclampsia and the susceptibility of podocytes [19]. Finally, the review summarizes the role of Toll-like receptors in the pathogenesis of glomerulopathy and their role as potential marker molecules for the development of renal diseases [20].

## References

[1] Andrade-Oliveira V., Foresto-Neto O., Watanabe I.K.M., Zatz R., Câmara N.O.S. (2019). Inflammation in Renal Diseases: New and Old Players. Front. Pharmacol..

[2] Baer P.C., Koch B., Geiger H. (2020). Kidney Inflammation, Injury and Regeneration. Int. J. Mol. Sci..

[3] Urbschat A., Thiemens A.-K., Mertens C., Rehwald C., Meier J.K., Baer P.C., Jung M. (2020). Macrophage-Secreted Lipocalin-2 Promotes Regeneration of Injured Primary Murine Renal Tubular Epithelial Cells. Int. J. Mol. Sci..

[4] Guiteras J., De Ramon L., Crespo E., Bolaños N., Barcelo-Batllori S., Martinez-Valenzuela L., Fontova P., Jarque M., Torija A., Bestard O. (2021). Dual and Opposite Costimulatory Targeting with a Novel Human Fusion Recombinant Protein Effectively Prevents Renal Warm Ischemia Reperfusion Injury and Allograft Rejection in Murine Models. Int. J. Mol. Sci..

[5] Nežić L., Škrbić R., Amidžić L., Gajanin R., Milovanović Z., Nepovimova E., Kuča K., Jaćević V. (2020). Protective Effects of Simvastatin on Endotoxin-Induced Acute Kidney Injury through Activation of Tubular Epithelial Cells’ Survival and Hindering Cytochrome C-Mediated Apoptosis. Int. J. Mol. Sci..

[6] Decuypere J.-P., Hutchinson S., Monbaliu D., Martinet W., Pirenne J., Jochmans I. (2020). Autophagy Dynamics and Modulation in a Rat Model of Renal Ischemia-Reperfusion Injury. Int. J. Mol. Sci..

[7] Moschovaki-Filippidou F., Steiger S., Lorenz G., Schmaderer C., Ribeiro A., Von Rauchhaupt E., Cohen C.D., Anders H.-J., Lindenmeyer M., Lech M. (2020). Growth Differentiation Factor 15 Ameliorates Anti-Glomerular Basement Membrane Glomerulonephritis in Mice. Int. J. Mol. Sci..

[8] Jung H.-Y., Oh S.-H., Ahn J.-S., Oh E.-J., Kim Y.-J., Kim C.-D., Park S.-H., Kim Y.-L., Cho J.-H. (2020). NOX1 Inhibition Attenuates Kidney Ischemia-Reperfusion Injury via Inhibition of ROS-Mediated ERK Signaling. Int. J. Mol. Sci..

[9] Leong K.G., Ozols E., Kanellis J., Nikolic-Paterson D.J., Ma F.Y. (2020). Cyclophilin A Promotes Inflammation in Acute Kidney Injury but Not in Renal Fibrosis. Int. J. Mol. Sci..

[10] Hong Y.A., Jung S.Y., Yang K.J., Im D.S., Jeong K.H., Park C.W., Hwang H.S. (2020). Cilastatin Preconditioning Attenuates Renal Ischemia-Reperfusion Injury via Hypoxia Inducible Factor-1α Activation. Int. J. Mol. Sci..

[11] Son M., Oh S., Choi J., Jang J., Son K., Byun K. (2021). Attenuating Effects of Dieckol on Hypertensive Nephropathy in Spontaneously Hypertensive Rats. Int. J. Mol. Sci..

[12] Jin J., Wang T., Park W., Li W., Kim W., Park S., Kang K. (2020). Inhibition of Yes-Associated Protein by Verteporfin Ameliorates Unilateral Ureteral Obstruction-Induced Renal Tubulointerstitial Inflammation and Fibrosis. Int. J. Mol. Sci..

[13] Kim Y.-J., Oh S.-H., Ahn J.-S., Yook J.-M., Kim C.-D., Park S.-H., Cho J.-H., Kim Y.-L. (2020). The Crucial Role of Xanthine Oxidase in CKD Progression Associated with Hypercholesterolemia. Int. J. Mol. Sci..

[14] De Miguel C., Kraus A.C., Saludes M.A., Konkalmatt P., Domínguez A.R., Asico L.D., Latham P.S., Offen D., Jose P.A., Cuevas S. (2020). ND-13, a DJ-1-Derived Peptide, Attenuates the Renal Expression of Fibrotic and Inflammatory Markers Associated with Unilateral Ureter Obstruction. Int. J. Mol. Sci..

[15] Steines L., Poth H., Herrmann M., Schuster A., Banas B., Bergler T. (2020). B Cell Activating Factor (BAFF) Is Required for the Development of Intra-Renal Tertiary Lymphoid Organs in Experimental Kidney Transplantation in Rats. Int. J. Mol. Sci..

[16] Maixnerova D., Tesar V. (2020). Emerging Modes of Treatment of IgA Nephropathy. Int. J. Mol. Sci..

[17] Nalewajska M., Gurazda K., Marchelek-Myśliwiec M., Pawlik A., Dziedziejko V. (2020). The Role of Endocan in Selected Kidney Diseases. Int. J. Mol. Sci..

[18] Gluba-Brzózka A., Franczyk B., Olszewski R., Rysz J. (2020). The Influence of Inflammation on Anemia in CKD Patients. Int. J. Mol. Sci..

[19] Kwiatkowska E., Stefańska K., Zieliński M., Sakowska J., Jankowiak M., Trzonkowski P., Marek-Trzonkowska N., Kwiatkowski S. (2020). Podocytes—The Most Vulnerable Renal Cells in Preeclampsia. Int. J. Mol. Sci..

[20] Mertowski S., Lipa P., Morawska I., Niedźwiedzka-Rystwej P., Bębnowska D., Hrynkiewicz R., Grywalska E., Roliński J., Załuska W. (2020). Toll-Like Receptor as a Potential Biomarker in Renal Diseases. Int. J. Mol. Sci..

